# The ‘Outcome Reporting in Brief Intervention Trials: Alcohol’ (ORBITAL) framework: protocol to determine a core outcome set for efficacy and effectiveness trials of alcohol screening and brief intervention

**DOI:** 10.1186/s13063-017-2335-3

**Published:** 2017-12-22

**Authors:** G. W. Shorter, N. Heather, Jeremy W. Bray, E. L. Giles, A. Holloway, C. Barbosa, A. H. Berman, A. J. O’Donnell, M. Clarke, K. J. Stockdale, D. Newbury-Birch

**Affiliations:** 1 Alcohol and Public Health Research Team, School of Health and Social Care, Teesside University, Middlesbrough, UK; 2 Psychotraumatology, Mental Health & Suicidal Behaviour Research Group, Psychology Research Institute, Ulster University, Coleraine, UK; 3 Inspire, Belfast, UK; 4 College of Medicine, Biology and Environment, Australian National University, Canberra, ACT Australia; 5 Faculty of Health and Life Science, Northumbria University, Newcastle upon Tyne, UK; 6 Department of Economics, Bryan School of Business and Economics, University of North Carolina at Greensboro, Greensboro, NC USA; 7 School of Health in Social Science, University of Edinburgh, Edinburgh, UK; 8 Behavioral Health Economics Program, RTI International, Chicago, IL USA; 9 Centre for Psychiatry Research, Department of Clinical Neuroscience, Karolinska Institutet, Stockholm, Sweden; 10 Stockholm Health Care Services, Stockholm County Council, Stockholm, Sweden; 11 Institute of Health and Society, Newcastle University, Newcastle Upon Tyne, UK; 12 Northern Ireland Methodology Hub, Queen’s University of Belfast, Belfast, UK; 13 School of Psychological and Social Sciences, York St. John University, York, UK

**Keywords:** Consensus methods, Core outcome set, Delphi technique, Screening, Brief intervention, Alcohol, Measurement, Alcohol brief intervention, Trial, Outcomes standardisation

## Abstract

**Background:**

The evidence base to assess the efficacy and effectiveness of alcohol brief interventions (ABI) is weakened by variation in the outcomes measured and by inconsistent reporting. The *‘*Outcome Reporting in Brief Intervention Trials: Alcohol’ (ORBITAL) project aims to develop a core outcome set (COS) and reporting guidance for its use in future trials of ABI in a range of settings.

**Methods/design:**

An international Special Interest Group was convened through INEBRIA (International Network on Brief Interventions for Alcohol and Other Drugs) to inform the development of a COS for trials of ABI. ORBITAL will incorporate a systematic review to map outcomes used in efficacy and effectiveness trials of ABI and their measurement properties, using the COnsensus-based Standards for the selection of health Measurement INstruments (COSMIN) criteria. This will support a multi-round Delphi study to prioritise outcomes. Delphi panellists will be drawn from a range of settings and stakeholder groups, and the Delphi study will also be used to determine if a single COS is relevant for all settings. A consensus meeting with key stakeholder representation will determine the final COS and associated guidance for its use in trials of ABI.

**Discussion:**

ORBITAL will develop a COS for alcohol screening and brief intervention trials, with outcomes stratified into domains and guidance on outcome measurement instruments. The standardisation of ABI outcomes and their measurement will support the ongoing development of ABI studies and a systematic synthesis of emerging research findings. We will track the extent to which the COS delivers on this promise through an exploration of the use of the guidance in the decade following COS publication.

## Background

As early as the start of the ‘WHO Collaborative Project on Detection and Management of Alcohol-related Problems in Primary Health Care’ in the early 1980s [1], researchers have recognised a need to address alcohol problems across a wide spectrum of use levels from hazardous use to dependence. Alcohol brief interventions (ABIs) have emerged as the main approach to addressing hazardous and harmful alcohol use in primary health care, beginning with the results of the DRAMS trial reported in 1987 [2]. However, ABIs can be used in a range of settings beyond primary care such as emergency departments [3, 4], general hospital wards [5, 6], schools [7, 8], online [9, 10], criminal justice [11, 12], and workplaces [13, 14].

ABIs vary in nature, ranging from very brief to more extended models and can be delivered in a variety of ways [15–17]. For the research presented here, we use National Institute for Health and Care Excellence (NICE) guidance PH24 [18] on the prevention and early identification of alcohol-use disorders (including hazardous drinking, harmful drinking and alcohol dependence) among adults and adolescents (aged 16 + years) to guide our definition of ABI. ABIs are defined as interventions suitable for those not seeking treatment for an alcohol problem but who are identified by screening as having, or being at risk of, problems from their alcohol use. ABIs are behavioural interventions that aim to help someone reduce their alcohol consumption. They can consist of short, often single sessions of feedback and tailored advice (brief advice), or longer, motivationally based interventions that explore motivations for drinking and personal barriers to change (extended brief intervention). PH24 recommends screening and brief intervention for people aged 16 and above who are at risk of, or experiencing, harm from alcohol and who are not engaged in, or seeking treatment for, alcohol dependence or alcohol-related problems. Although ABI have been used by some in treatment contexts e.g. [19] we use the PH24 definition.

Multiple systematic reviews and meta-analyses support the effectiveness of ABIs in reducing alcohol use in primary health care [20–22]. However, research on ABI is marked by conflicting findings regarding efficacy and effectiveness across primary health care, emergency services, hospitals, criminal justice, workplaces, online, and other settings [23–29]. An avoidable source of this conflict arises from the wide range of outcomes used to measure alcohol-related and other relevant change. The use of these different endpoints poses a challenge to combining data in meta-analysis to ascertain efficacy and effectiveness, and contribute to waste in research [30]. The lack of consistency and standardisation in outcomes also impedes the conduct of economic evaluations in alcohol research [31]. The selection and application of well-designed outcomes by trial steering committees or other evaluation researchers [32] is relevant to all stakeholders including service users, practitioners and policymakers. Optimised measurement of change will maximise the potential of ABI research to influence decision-making, as it has in other research areas such as rheumatoid arthritis [33, 34].

Systematic reviews summarising the effectiveness of a wide range of health care interventions shows considerable heterogeneity in outcome measures used [35], and research has also highlighted the problem of selective and incomplete reporting [36] in Cochrane reviews. The same is true for reviews of ABI delivered in a variety of settings [20, 37–42]. As such, it is difficult to compare findings of different research trials and other evaluations, even though they aim to address similar research questions. As replicability is essential to scientific progress [43], the use of different instruments measuring key outcomes in studies testing the same general hypotheses impedes progress in the ABI field. Furthermore, if we aim for improved change measurement, a reliance on statistical significance should be replaced by precise estimates of differences, which is again dependent on the use of common measures to compare effect sizes. Meta-analysis is more accurate and informative when applied to a common metric rather than a generalised effect size convention [44].

Both the Consolidated Standards of Reporting Trials (CONSORT) Statement [45] and the Standard Protocol Items: Recommendations for Interventional Trials (SPIRIT) Statement [46] recommend the use of a core outcome set (COS), and draw upon the COMET Initiative for guidance [35]. Developing a standard way for authors to prepare and present trial findings ensures complete and transparent reporting, which is essential to critical appraisal and interpretation.

At the workshop ‘Design and conduct of randomised controlled trials of brief interventions for alcohol and drugs’ at the INEBRIA conference in Warsaw in 2014, it was proposed to establish a Special Interest Group (SIG) to consider ways to standardise measures used in research on the efficacy and effectiveness of brief interventions. This proposal was approved by the INEBRIA Coordinating Committee, leading to the formation of the INEBRIA Research Measurement Standardisation Group and, later, the Outcome Reporting in Brief Intervention Trials (ORBITAL) Group which, as of 2016, includes 29 members.

### Aims and research questions

The primary aim of ORBITAL is to develop a COS for ABI efficacy and effectiveness trials. The secondary aim is to understand the variability in outcomes and measurement instruments in existing trials across all settings and to organise this information in the most efficient way for future trials of ABI in a published statement, the ‘ORBITAL Statement’.

The research questions are:

1. What domains, outcomes, and instruments have been used in efficacy and effectiveness trials of ABI?
2. What outcomes are ranked by stakeholders as most important for the ORBITAL COS?
3. What outcomes are relevant for different settings?
4. To what extent is the COS used in the decade following its publication?

## Methods/design

The first step in developing this COS will be to review existing academic literature to identify outcomes organised by domain (‘what to measure’) and instruments (‘how to measure’). Instruments will be rated in terms of validity, reliability, responsiveness to change and usability in ABI settings, effectively summarising and mapping change measurement in the ABI field. The ratings will be based on the COSMIN criteria [47]. This map of change measurement will be presented to stakeholders in the field, including patient and public representatives (PPR) and beneficiaries of ABI. For this study, PPR are advocates for those who are experiencing alcohol problems, hazardous or harmful drinkers, and those with prior experience of dependence, some of whom may have used an ABI in the past. This represents the range of end users of ABIs. Priority outcomes will be identified through Delphi consensus. The findings will be discussed and incorporated in the ORBITAL Statement of reporting and measurement guidance on a COS organised into domains. Given the breadth of the settings in which ABIs are implemented, the structure and content of the final ORBITAL Statement is yet to be determined since the COS might contain outcomes and instruments that are applicable across all settings and content areas, or there might be a core with a subset of outcomes and instruments unique to a single setting or to specific content areas. The Delphi study and consensus meeting will incorporate this decision-making element, and the decision-making process will be carefully documented and reviewed for potential bias.

The methods of this project comprise five phases. Phase 1 consists of a systematic review to identify the outcomes and instruments (ranked by COSMIN criteria). Full details of this review can be found in PROSPERO (registration number: CRD42016047185) and will not be discussed further here. The second to fifth phases, discussed below, are: (2) a Delphi study to rank the outcomes in priority order; (3) a consensus meeting to determine the final set and instruments; (4) reporting and dissemination; and (5) an assessment of the utility of the COS in the decade following the publication of the ORBITAL COS Statement.

### Phase 2: Delphi study

To reduce the number of possible outcomes to a priority list for discussion at a consensus meeting, we will use the Delphi method. This is an iterative consensus technique which comprises sequential questionnaires answered anonymously by a range of relevant participants [48]. We will seek input from an international and diverse stakeholder panel. A series of sequential questionnaires will be presented in ‘rounds’. The Delphi panel will comprise a range of stakeholders recruited from the following groups who will be contacted directly by email, with the suggestion to share the invitation to participate with their colleagues. The stakeholders (some of which overlap) include:

- Trial investigators – first and last authors of reports of randomised trials in the field identified in the systematic review
- Members of the INEBRIA SIG of the ORBITAL project (which includes researchers in brief interventions, methodologists, trialists, and health professionals)
- Executive leadership of relevant scientific organisations such as the Research Society on Alcoholism, the Association for Medical Education and Research in Substance Abuse, the Society for the Study of Addiction to Alcohol and Other Drugs, and the College on Problems of Drug Dependence
- Cochrane Review Group on Drugs and Alcohol
- NICE Alcohol-use Disorders: Prevention PH24 Membership Group
- People listed as being involved in ABI clinical guidance including through the World Health Organisation (WHO), the US Centers for Disease Control and Prevention (CDC), the US Substance Abuse and Mental Health Services Administration (SAMHSA), the US National Institutes of Health (NIH), and the National Board of Health and Welfare (Sweden)
- Research funders acknowledged as funding the ABI studies included in the review
- Journal editors and the Editorial Boards of journals who are members of the International Society of Addiction Journal Editors
- ABI academics who have experience of being on Research Ethics Committees
- Statisticians, trialists, and COS developers
- Service users and service-user representatives
- Practitioners: including representatives from health, education, alcohol and drug services, criminal justice, and workplace health

#### Panel size and recruitment

There are no accepted guidelines for panel size to achieve stable consensus in a Delphi analysis. Like others [49], we will be guided by practicality, scope, and time available. We will aim to recruit and retain as large a panel as possible. Participants will be invited to nominate others to contribute (snowball sampling). Individuals will be identified through a range of contacts by ORBITAL team members, including INEBRIA members. The project manager (GS) and other members of the Steering Group will invite participants from international stakeholder groups, using distribution lists such as *Addict-L, Addiction Medicine, Drug Misuse Research, BALANCE, Alcohol Misuse* (Scottish Addiction Studies), *Drugtalk, Ewodor,* and the *Kettil Bruun Society.* Other key methodologists will be reached through the UK Hubs for Trials Methodology Research and Clinical Trials Units in the UK. We will also recruit Delphi panellists from the WHO Department of Mental Health and Substance Abuse and from the US NIH, CDC, and SAMHSA.

We aim to complete the Delphi analysis after two rounds. However, if deemed necessary by the ORBITAL Steering Group because of a clear lack of consensus we will have a third round. We will reduce attrition between rounds through the following techniques: a personalised invitation, outline of timelines, personalised email reminders (every 2 weeks, but with no more than four reminders), and provision of an easy interface which minimises the time required to complete each round [49]. We will use online COMET software designed specifically for the purposes of conducting Delphi studies to generate COS. The importance of completing all rounds will be emphasised to participants in the Information Sheet.

#### The Delphi questionnaire and rounds

In round 1, participants will be asked to rate each outcome using a scale of 1–9, with 1–3 labelled ‘not important for inclusion’, 4–6 labelled ‘important but not critical’ and 7–9 labelled ‘critical for inclusion’ [49]. The outcomes will be generated from the systematic review in phase 1. Outcomes will be listed randomly, and hosted on COMET software (DelphiManager), to avoid leading participants to believe that some are more important than others. PPR will be consulted in the presentation and explanation of the outcomes. Participants also have the opportunity to add additional outcomes and to comment on why they have ranked outcomes as they have. Novel outcomes will be reviewed and coded by at least two reviewers to determine if they are indeed novel and cannot be subsumed into existing outcomes; where there is disagreement, an additional reviewer will be consulted.

All outcomes will be carried through to the second round with first round scores displayed for each outcome calculated as a percentage of responses split by respondent group. Consensus is defined a priori as 70% or more of the respondents scoring an outcome from 7 to 9 and fewer than 15% scoring it 1 to 3, in line with similar studies [48–51]. All other score distributions would be taken to indicate lack of agreement for inclusion of a given outcome in the COS. The rationale for this threshold reflects an outcome agreed by the majority as critically important, with only a small minority ranking an outcome as being of little or no importance. Likewise, consensus that an outcome should not be in the COS requires agreement by the majority that the outcome is of little or no importance, with no more than a small minority considering it to be critically important. We recognise that choice of thresholds is somewhat subjective, but specification of the definition in a study protocol reduces the risk that researchers will define consensus post hoc in a way that would bias the conclusions toward their own beliefs [35].

#### Ethical considerations

Ethics approval will be sought from Teeside University Ethics Committee to conduct the Delphi study. Informed consent will be sought from all participants. Individual participants will be approached or will volunteer themselves by the methods outlined above. They will be sent a short email introducing the study, with more information on the study and consent forms available online. Information on the composition of the Delphi panel will be collated, including age, background, experience in years, field of interest, and current working location. Participants in the Delphi panel who agree to be named will be listed in alphabetical order online, and participants who request it will be emailed the final ORBITAL Statement.

### Phase 3: consensus meeting

After the Delphi study is completed, a convenience sample of representatives of the stakeholder groups will be invited to join a consensus meeting to determine the final COS. We will aim to include at least one person from each stakeholder group, and understand that there may be overlap. This is in keeping with guidance from Tugwell et al. [30] and Prinsen et al. [34, 52]. The consensus meeting will be recorded to evidence decision-making. The meeting minutes will be available, and a summary report will be published. The results of the Delphi study on what to measure will be discussed at the consensus meeting and representatives will be asked to discuss ‘how’ the outcome variables in the COS should be measured. If the number of outcomes prioritised in the Delphi study is too large to allow time to discuss the measurement of the COS items during the main consensus meeting, a separate consensus meeting may be held by teleconference to consider the COSMIN rankings of each measure under each outcome. Given that the number of domains will be unknown until the Delphi is complete, a second Delphi may also be proposed to rank the ‘how’ to measure under each of the domains in the event that (1) there is limited evidence to support ‘how’ to measure domains or (2) there are too many measures under each domain to discuss at a consensus meeting. Should this be required, a separate protocol will be generated, and a separate ethics application sought from Teeside University Ethics Committee. The editors from key addiction journals will be invited to discuss how best to use and disseminate the COS.

### Phase 4: reporting and dissemination

The protocol for the ORBITAL COS has been registered on the COMET Initiative website to prevent unnecessary duplication of effort. Several subsequent ORBITAL publications are planned, including the results of the systematic review to identify what outcomes to measure and the COSMIN ranking of how to measure them. The Delphi study will also be written up for publication, noting the changes to the list of outcomes suggested at each stage. The consensus meeting will be documented and a manuscript written describing any decision-making challenges and how these were overcome. The papers relating to the ORBITAL COS statment will be reported using the COS-STAR guidance [53].

### Phase 5: assessment of the uptake of the COS in future trials

Subject to funding, regular checks on how the COS is used over the subsequent decade will be carried out using Google Scholar and Scopus. Alerts will be set up to notify the team when the COS is cited and annual updates will be reported to the INEBRIA Coordinating Committee and the INEBRIA general membership at the annual conference and via the INEBRIA Google Group.

## Discussion

This protocol describes a programme of work to develop a COS for assessing the efficacy and effectiveness of ABI in clinical trials across all settings. The scope for the COS is deliberately broad, given the wide variety of locations and types of ABI. This in itself is a challenge, and the nature of the COS will depend greatly on the findings of the systematic review. To the best of our knowledge, this will be the first Delphi study to ask for comment on the suitability of outcomes across such a wide range of settings, and to raise the possibility of having a COS which is based around a core subset with some sub-core aspects depending on the setting or nature of the brief intervention. As such, the decision-making process in developing the COS will need to be clear on how and why decisions were made and by whom [48]. Other innovative features of this protocol include the plan to follow up on the utility and use of the COS and an a priori recognition that technological developments and changes in health care worldwide will make it important to revisit the COS periodically, assessing its continued utility for the contemporary ABI field.

## Study status

As of April 2017, the systematic review (PROSPERO registration number: CRD42016047185) has started. The Delphi study has not yet begun, and a list of potential participants is being collated.
